# Sown Wildflowers Enhance Habitats of Pollinators and Beneficial Arthropods in a Tomato Field Margin

**DOI:** 10.3390/plants10051003

**Published:** 2021-05-17

**Authors:** Vaya Kati, Filitsa Karamaouna, Leonidas Economou, Photini V. Mylona, Maria Samara, Mircea-Dan Mitroiu, Myrto Barda, Mike Edwards, Sofia Liberopoulou

**Affiliations:** 1 Scientific Directorate of Pesticides Control and Phytopharmacy, Benaki Phytopathological Institute, 8 Stefanou Delta Str., 14561 Kifissia, Greece; l.economou@bpi.gr (L.E.); m.samara@bpi.gr (M.S.); m.barda@bpi.gr (M.B.); albilyb@hotmail.com (S.L.); 2 HAO-DEMETER, Institute of Plant Breeding & Genetic Resources, 57001 Thessaloniki, Greece; phmylona@nagref.gr; 3 Faculty of Biology, Alexandru Ioan Cuza University, Bd. Carol I 20A, 700505 Iași, Romania; mircea.mitroiu@uaic.ro; 4 Mike Edwards Ecological and Data Services Ltd., Midhurst GU29 9NQ, UK; ammophila@macace.net

**Keywords:** field margin, flowering plants, Hymenoptera pollinators, beneficial arthropods, crop pollination, processing tomato

## Abstract

We evaluated the capacity of selected plants, sown along a processing tomato field margin in central Greece and natural vegetation, to attract beneficial and Hymenoptera pollinating insects and questioned whether they can distract pollinators from crop flowers. Measurements of flower cover and attracted pollinators and beneficial arthropods were recorded from early-May to mid-July, during the cultivation period of the crop. Flower cover was higher in the sown mixtures compared to natural vegetation and was positively correlated with the number of attracted pollinators. The sown *Glebionis coronaria*, *Coriandrum sativum*, *Anethum graveolens*, and *Fagopyrum esculentum* attracted mainly wild bees, which were the most abundant pollinating insects. In the natural vegetation, *Rapistrum rugosum* attracted mainly honeybees, while Asteraceae, Convolvulaceae, and Apiaceae species attracted wild bees. Beneficial arthropod abundance and diversity were higher in the sown mixture. Tomato flowers were visited by a small number of wild bees. Their number was not affected by the distance from the field margin, indicating no distraction effect from the sown or natural vegetation flowering plants. Our results suggest that selected flowering plants can improve the field margin habitats for pollinating insects and beneficial arthropods, but more work is needed to elucidate the effect on crop pollination.

## 1. Introduction

The increasing global demand for food has pushed modern farming systems into setting higher crop productivity goals, leading to intensive cultivation practices [1] and the simplification of the agricultural landscape, with vast monocultures and an over-reliance on agrochemical inputs. However, these practices have been associated with several negative consequences on the functioning of agroecosystems, especially in arable farmland [2]. Intensive cultivation practices in arable fields have led to the deterioration of flora and fauna biodiversity [3], threatening the sustainability of crop production and ultimately undermine the very same goal of food security they were employed to achieve. Among the main agroecosystem services affected is crop pollination, essential for the productivity of 75% of the main food crops [4] with an estimated worldwide value of 153 € billion [5] and biological pest control, with an estimated global value of around 40 € to 74 € billion [6]. Thus, understanding the negative impacts of agricultural intensification on crop productivity and developing sustainable management strategies to offset them, has increasingly been in the spotlight of research [7,8,9]. Several studies have addressed the loss of habitats for pollinating insects and beneficial arthropods in intensive farming systems and the associated decline in pollination [10,11] and biological control services [12,13], while others outlined the complex, mutually beneficial interaction between weeds and insect pollinators in the agricultural landscape [14,15]. 

Many European countries, especially central and north, have widely adopted the practice of sowing selected plant species to hinder the deterioration of habitats for pollinators and beneficial arthropods and support the interrelated multitrophic systems [16]. Purpose-designed schemes, providing economic incentives for farmers to implement agro-environmental measures, such as appropriate management of field margins, have contributed to the wide implementation of these practices [2,17,18]. Plant mixtures, comprising annual and perennial species, have been studied for their role in supporting pollinators, beneficial arthropods, and other invertebrates in various crops and landscape scenarios [19,20,21]. However, the practice of field margin management is not commonplace in southern European countries, like Greece, where the small field size, measuring on average 6.6 ha [22] means that, to maximise economic returns, the available land is cultivated from end to end. Consequently, the information on flora and fauna biodiversity and their ecosystem services is limited and is available only for the perennial olive orchards [23,24,25]. 

Field grown tomato (*Solanum lycopersicum*) is self-pollinated or partially dependent on flower-visiting insects for effective pollination and fruit production [4]. Knowledge on the contribution of insects, especially wild bees, for the pollination of processing tomato remains limited [26]. Greenleaf and Kremen [26] demonstrated that, although the field-grown tomato is regarded as mainly self-pollinated, its production was substantially increased under pollination by wild bees such as *Bombus* and *Anthophora* species, and suggested that cross-pollination may enhance tomato fruit-set over self-pollination, especially in tomatoes grown under high temperatures which can cause pollen sterility. The exent of this phenomenon is variable and depends on the position of the flowers on the plant, thus cross-pollination can increase the chances of fertile pollen reaching more flowers [26]. According to Teppner [27], pollinators on open cultivated tomato in Central Europe, which benefit the pollination of tomato due to their ‘buzzing’ vibration effect on the flowers, included the *Bombus* species *B. pascuorum* and *B. terrestris*, *Megachile willughbiela*, *Hylaeus gibbus* and buzzing *Lasioglossum* species. Therefore, bees associated with the pollination of field grown tomato are important for crop production and their communities should be maintained with the support of suitable habitats that provide food and shelter [26]. Moreover, the presence of non-crop flowering plant habitats could also contribute to the conservation of parasitoids and generalist natural enemies that ultimately could suppress tomato pests and reduce crop damage [19,28,29,30]. None the less, field margin plants and arthropod communities’ interaction is complex and can vary greatly depending on plant species composition [31]. For example, some plant species could provide resources not only to natural enemies but also to pests, shifting the balance towards herbivorous populations and eventually leading to crop damage [32]. In addition, plant species which are attractive to natural enemies or pollinating insects could act either as a reservoir that would benefit the crop [33] or as a distraction for these insects from the crop plants. 

Here, we questioned whether selected flowering species sown in the field margin of a processing tomato located in one of the main areas for this crop in Greece, can establish successfully and create a habitat that would increase the abundance and diversity of pollinating insects and beneficial arthropods in the field margin compared to natural vegetation of arable weeds and examined whether this practice could have a distraction effect for the pollinators visiting crop plants. The current study was conducted in the frame of the biodiversity project Operation Pollinator, implemented in various crops in Greece since 2010 [23]. The aims of the project follow the principles of the EU policy for sustainable agriculture, dictated through the new common agriculture policy (CAP) and the Directive (2009/128/EC) for the sustainable use of plant protection products, as it supports the protection and enhancement of flora and fauna biodiversity in the agro-ecosystems.

## 2. Results

### 2.1. Sown Plants Establishment and Flowering

**Figure 1:**
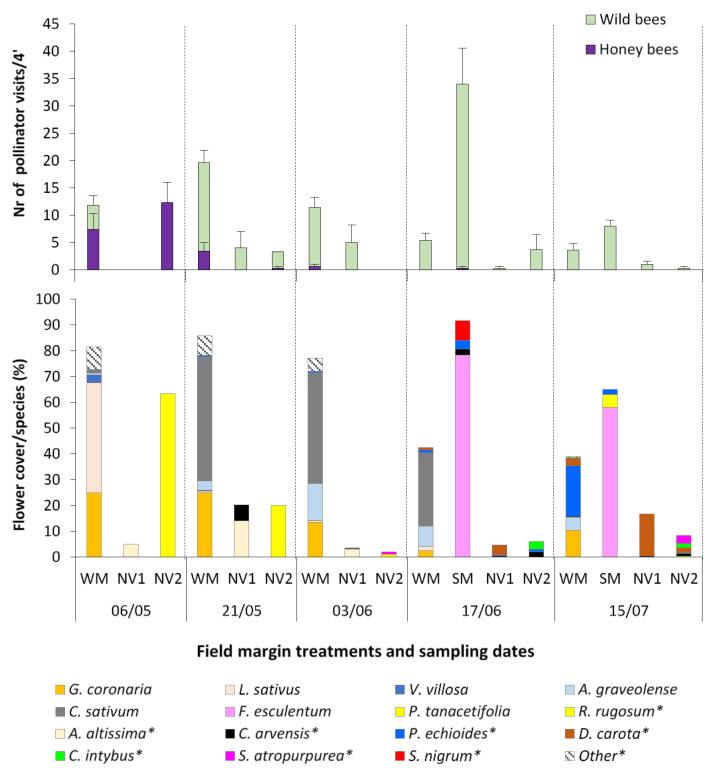
Mean flower cover percentage per species in the winter mixture (WM), spring mixture (SM) and two natural vegetation sites (NV1 and NV2), and corresponding mean numbers of Hymenoptera pollinator visits (wild bees and honey bees) recorded for 4′/14 m^2^ plot, at five sampling dates. Flowering species in the natural vegetation and flowering weeds that emerged in the plots with the sown mixtures are noted with an asterisk (*). Other species: *Sonchus* sp., *S. arvensis*, *P. rhoeas*, *L. serriola*, *V. hederifolia*, *Lathyrus* sp. Vertical bars represent standard error of means.

Flowering of the winter mixture (WM) started around mid-April and lasted until mid-July (Figure 1 and Supplementary Figure S1). Species reached flowering in the successive order of *Vicia villosa* = *Lathyrus sativus* > *Glebionis coronaria* > *Coriandrum sativum* > *Anethum graveolens*. The emergence of the remaining sown species, *Tagetes patula* and *Matricaria chamomilla*, was in small numbers (<1 plant/m^2^) and their flowering had negligible contribution. The Fabaceae species were the earliest to reach flowering which lasted until early May, while *G. coronaria*, which started flowering around early-May, had the longest lasting flowering period (more than eight weeks with a peak towards the end of May) compared to all the other species. Both sown Apiaceae species started flowering around mid-May. Arable weeds that emerged together with the sown ones in WM included the species *Sonchus oleraceous*, *Picris echioides*, *Lactuca serriola*, *Cichorium intybus*, *Veronica hederifolia*, *Sinapis arvensis*, *Papaver rhoeas*, *Daucus carota*, and *Convolvulus arvensis*. A two-way ANOVA was carried out on the % flower cover by type of winter treatment (sown WM and natural vegetation sites NV1 and NV2) and time (five sampling dates, from May to July) (Supplementary Table S1). The main effects of treatment type and time, and their interaction were significant. Overall, the sown WM had significantly higher flower cover than either the NV1 or NV2 site. The difference was significant from late-May to mid-July, while in early-May the flower cover between the WM and the NV2 site was similar.

In the summer mixture (SM), *Fagopyrum esculentum* established successfully and reached flowering from mid-June to mid-July, while *Phacelia tanacetifolia* emerged in small numbers and had limited contribution to the percentage of flower cover (Figure 1). *Petroselinum crispum* did not emerge. The SM plots had also a low density of arable weeds (dicotyledons *Convolvulus arvensis*, *Picris echioides*, *Solanum nigrum*, *Abutilon theophrasti*, *Amaranthus* sp., and monocotyledons *Sorghum halepense*, *Echinochloa crus-galli*, *Digitaria* sp., and *Cyperus* sp.). A two-way ANOVA was carried out on the % flower cover by type of field margin treatment (including both sown mixtures SM and WM and the natural vegetation sites NV1 and NV2) and time (two sampling dates in mid-June and mid-July, when SM and WM had synchronous flowering). The main effect of treatment was significant, while the effect of time and their interaction was not significant (Supplementary Table S1). The SM had significantly higher flower cover compared to either of the NV sites and the WM.

The flowering in the two natural vegetation sites NV1 and NV2 was provided by different species (Supplementary Figure S2). These mainly were *Picris echioides*, *Daucus carota*, *Anthemis altissima*, and *Convolvulus arvensis* in the NV1 site, and *Rapistrum rugosum*, *Scabiosa atropurpurea*, *Convolvulus arvensis*, and *Cichorium intybus* in the NV2 site (Figure 1). Other flowering species emerging outside the designated NV plots, such as *Matricaria chamomilla* (Asteraceae) early in the season and *Galega officinalis* (Fabaceae) at the banks of a neighboring draining channel in July, were recorded, though not included in the measurements.

The tomato crop was in flower from June to July and was harvested at the end of July. The economic crop flowering, i.e., the flowers that produced the fruits harvested, were those pollinated before mid-July, while any fruits produced after that period were not of economic value for the farmer (they were not harvested). Therefore, the economic crop flowering coincided with the end of flowering of the WM and the peak of flowering in the SM.

### 2.2. Effect of Wildflower Margins on Pollinator Abundance and Diversity

**Figure 2:**
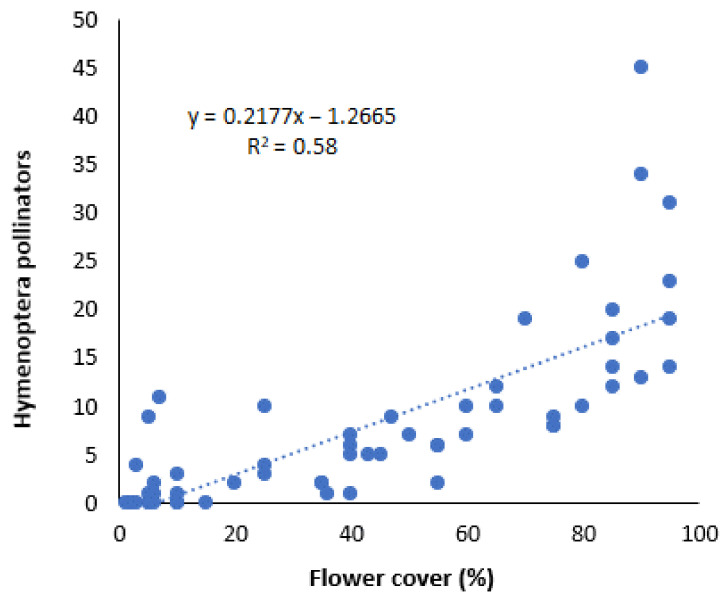
Correlation of flowering cover (%) in the field margins with sown or natural vegetation species and mean number of Hymenoptera pollinators (wild bees and honey bees) recorded at five sampling dates during May–July.

The Hymenoptera pollinators were honeybees early in the season and predominantly wild bees thereafter (Figure 1). Bumble bees or megachilids were not present at any sampling date. The total number of Hymenoptera pollinators visiting the flowers of the sown or the natural vegetation plants in the margins and the corresponding percentage of flower cover in each site had a positive correlation (R^2^ = 0.58) (Figure 2).

The type of treatment (WM or SM vs. natural vegetation margin) and the sampling time had a significant effect on the number of wild bees and pollinators in total (2-way ANOVA, *p* = 0.05; Supplementary Table S2) while their interaction was not significant. The WM field margin attracted significantly higher number of total pollinators (10 visits/plot/4′) compared to the NV1 and NV2 plots (two and four visits/plot/4′, respectively) while higher number of pollinators was recorded in late-May to early-June, compared to that in mid-June to mid-July, which is in line with the decline in flowering recorded after mid-June in the winter sown species (Figure 1 and Supplementary Table S2). The SM field margin also had significantly higher number of total pollinators (21 visits/plot/4′), compared to either the NV1 or NV2 site (0.3 and two visits/plot/4′, respectively), with significantly more visits recorded in mid-June compared to mid-July (Figure 1 and Supplementary Table S2). The number of wild bee visits recorded at the SM margin in mid-June was the largest recorded across all measurements in any margin type and sampling date (33.7 ± 6.6 visits/plot/4′). Wild bees were highly attracted by *F. esculentum*, which had a flowering peak at that time (Figure 1). 

The wild bees associated with the WM plots (*Andrena* spp., *Colletes* sp., *Hylaeus* spp., *Halictus* sp., *Lasioglossum* spp., *Pseudapis* sp., *Sphecodes* sp., and a few *Eucera* sp.) were observed to forage on *G.* *coronaria*, *C. sativum* and *A. graveolens*, throughout the flowering period of these species (Supplementary Table S3). *Fagopyrum esculentum* in the SM plots attracted mainly *Andrena* sp. and *Halictus* sp. (Supplementary Table S3). On the other hand, honeybees (*Apis mellifera*) were mainly recorded on the flowers of *L. sativus* in early-May and in smaller numbers on *C. sativum* later that month (Figure 1). The flowering plants in the NV plots attracted wild bees throughout all measurements, with the exception of *R. rugosum* in the NV2 plots, which was mainly foraged by honeybees when it was in full flower (early-May) (Figure 1). In late-May, towards the end of *R. rugosum* flowering, honeybee numbers dropped significantly, while the main pollinators recorded were small numbers of wild bees. Other flowering species of the NV plots that attracted wild bees were *Anthemis altissima* (Asteraceae), *Picris echioides* (Asteraceae), *Daucus carota* (Apiaceae) and *Scabiosa atropurpurea* (Dipsacaceae). The wild bee specimens associated with the NV plots included mainly *Andrena* spp.

The field margins attracted also non-Hymenoptera insects that could contribute generally to pollination. In the WM, these were Diptera (Syrphidae, Stratiomyidae (*Odontomyia* sp.)) and Lepidoptera on *G. coronaria*, and Diptera (Syrphidae) on *L. sativus*, and in the SM Diptera (mostly Syrphidae, other flies) and Lepidoptera on the flowers of *F. esculentum.* Finally, although not included in the measurements, it is noteworthy that *Galega officinalis* recorded in the natural vegetation outside the designated plots in mid-July, was the only flowering species that attracted *Bombus* sp.

The pollinators observed on the crop flowers during the two sampling days (17 and 23 June) were only wild bees, while no honeybees or other Apidae were present. The number of wild bees was not affected by the distance between the tomato crop row and the sown margin and remained very low (1–2 individuals in total of three replications, in each sampling site).

### 2.3. Effect of Wildflower Margins on Arthropod Abundance and Diversity

Suction sampling from the sown mixtures and the natural vegetation sites resulted in a total of 2256 individuals, belonging to over 50 families of Insecta and Arachnida taxa (Supplementary Table S4). Beneficial arthropods included mostly Hymenoptera parasitoids, generalist or specialist insect predators and spiders. The field margin with winter treatment (WM and NV) and sampling time had a significant effect on the Shannon diversity index (*H*) of total arthropods and Hymenoptera parasitoids (families) recorded in the suction samples (two-way ANOVA, *p* = 0.05; Table 1). The *H* index of total arthropods in the WM (2.26) was significantly higher compared to the native vegetation plots NV1 (1.90) and NV2 (2.04) (Table 1). On the other hand, the *H* index of the Hymenoptera parasitoids was significantly higher in the WM (1.34) compared to NV1 (0.93) but similar to NV2 (1.05), while it was significantly lower at the early-June measurement. Interestingly, despite the progressively lower flower cover of the WM with time, the *H* index of total arthropods remained significantly higher for this mixture (2.58) than the SM (2.19), with the latter being similar to the NV1 (2.21) and NV2 (2.19) sites (Table 1).

The main effect of field margin treatment (either for the winter treatments WM and NV1, NV2 or for both the winter and summer treatments WM, SM, NV1, NV2) and of time was significant on the abundance of Hymenoptera parasitoids and predators. The interaction between field margin type and time was not significant. The low numbers of parasitoids recorded in the WM plots at the early-June measurement compared to previous and later samplings, could be due to a possible temporary negative effect from pesticide application to the crop the day before the sampling.

**Figure 3:**
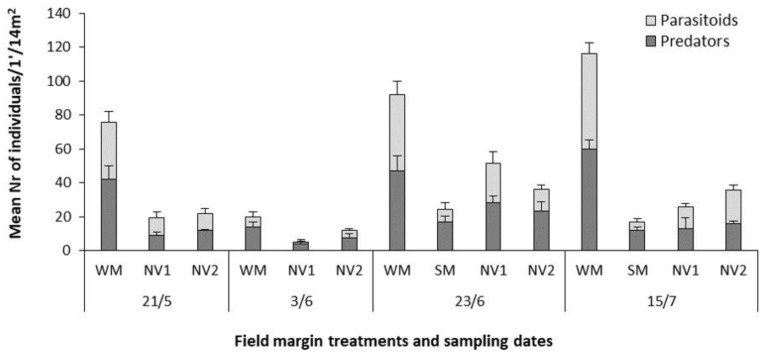
Mean number of parasitoids and predators recorded in suction samples (1′/14 m^2^ plot) from the sown (winter mixture WM, summer mixture SM) or natural vegetation (NV) field margins of a processing tomato crop. Vertical bars represent standard error of means.

The WM samples contained significantly more parasitoids and predators compared to any other field margin treatment (including SM) across all sampling times (Figure 3 and Supplementary Table S5). The overall mean (±s.e.m.) of natural enemies across the winter treatments was 35.1 (±5.2) parasitoids and 40.6 (±4.9) predators in the WM, while the corresponding means in the NV1 samples were 11.7 (±2.9) and 13.6 (±3.2) and in the NV2 were 11.6 (±2) and 14.7 (±2.2) for parasitoids and predators, respectively. The highest number of natural enemies was recorded in the WM at the mid-July measurement, with 56.4 (±6.8) parasitoids and 59.6 (±5.8) predators, respectively. At that time, despite coinciding with the peak of *F. esculentum* flowering, the samples from the SM plots contained only 4.7 (±2.2) parasitoids and 12 (±2.1) predators (Supplementary Table S5). Correlation analysis between the beneficial arthropod abundance and the percentage of flower cover revealed no association (R^2^ = 0.02). The relationship between the beneficial arthropods and the flowering species diversity (*H*) was more evident, although still weak (R^2^ = 0.18).

**Figure 4:**
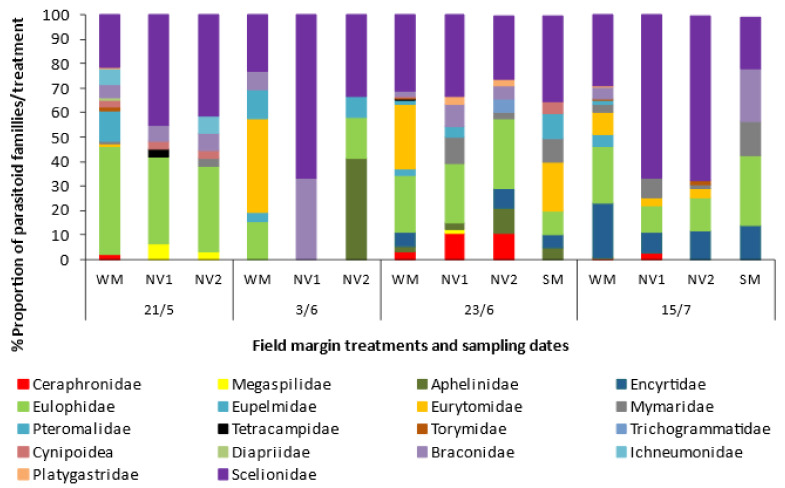
Percentage proportion of parasitoid families in samples of the winter mix (WM), summer mix (SM) and the two sites with natural vegetation (NV1 and NV2), at four sampling dates.

Parasitic wasps in the samples belonged to six different superfamilies (Ceraphronoidea, Chalcidoidea, Cynipoidea, Diaprioidea, Ichneumonoidea, Platygastroidea) and 18 different families (Figure 4 and Supplementary Table S6). The samples from the WM belonged to 17 families, while the ones from the SM mixture belonged to nine families and from the NV1 and NV2 sites to 13 and 15 families, respectively. Eulophidae and Scelionidae added to more than half of the samples in the WM (approx. 55.5%) while other abundant families were Eurytomidae and Encyrtidae. In the SM, Scelionidae held almost one third of the samples, followed by Eulophidae (17.6%), Eurytomidae (11.8%) and Mymaridae (11.8%). In the natural vegetation plots of both sites, Scelionidae was also the predominant family holding almost 50% of the samples and Eulophidae held another 22–23%. Aphelinidae, Braconidae, and Ichneumonidae, contributing lower percentages in the mixture or the natural vegetation samples, should not be overlooked as they are known families of aphid parasitoids, while other braconids and ichneumonids parasitize larvae of lepidopteran pests. Trichogrammatidae, known to include egg parasitoid species of Lepidoptera larvae, was recorded only on the NV2 plots in the late-June measurement. The sampled insect predators belong to the families Anthocoridae, Chrysopidae, Coccinellidae, Miridae, Syrphidae, and Reduviidae, with the latter found only at natural vegetation NV1 plots (Supplementary Table S4).

Regarding the presence of insect pests on the tomato crop, infestation by the cotton bollworm, *Helicoverpa armigera* (Lepidoptera: Noctuidae) was recorded after visual scouting during the crop season. Its occurrence was common in our experimentation field and within the usual incidence of the pest in the area.

## 3. Discussion

This work sheds light on the association of plant, pollinator, and beneficial arthropod communities in the field margin of processing tomato, a crop where similar studies are limited. Understanding these associations is the basis of manipulating habitats to augment the ecosystem functions of pollination and biological control. Several biodiversity-ecosystem function studies focus on the manipulation of species richness to measure the effect on an associated ecosystem function [34,35] while others argue that it is probably more important to understand the linkages between key species or functional groups and ecosystem functions [28,36]. The second approach has revealed, in many cases, some level of functional redundancy among species in the provision of certain ecosystem functions [28,37,38]. The selection of different flowering plants and their composition in mixtures to optimize the conservation of arthropod functional groups depends on the suitability of nectar and pollen resources they provide to these groups, on specific plant traits such as floral morphology associated with these groups, and on food preferences and foraging risks for flower visitors [39,40]. Flowering plant mixtures belonging to Apiaceae and other species with exposed floral nectaries are frequently used in biocontrol studies, while pollinator-friendly plant mixtures are often dominated by Fabaceae species [41]. 

Several studies have suggested that wildflower strips could benefit pollinator diversity [42] by comparing the effect with bare soil. In our study, a more challenging situation was tested as a control, i.e., natural vegetation in the field margins of a processing tomato crop. Although the sown mixtures had comparable or lower species diversity than the natural vegetation at the margins of the crop, they provided higher flower cover throughout the crop season and attracted more Hymenoptera pollinators. The tested WM provided a rich flowering canvas of *L. sativus*, *G. coronaria*, *C. sativum*, and *A. graveolens*, which changed in composition over time, lasting from early-May to mid-July and overlapped with the dense flowering provided mainly by *F. esculentum* in the SM. In terms of conservation of functional pollinator species, the abundance and relative ratio of wild bees and honeybees were associated with the flower species synthesis of the mixture over time, and outcompeted the natural vegetation. Among the attracted wild bee species, *Hylaeus* spp. and *Lasioglossum* spp. are known to pollinate tomato [27]. Several other wild bees were attracted, which, although not directly involved in the pollination of tomato, included species that are important for their pollination services to other plants of economic or biodiversity value. These included mainly *Andrena* spp., *Colletes* sp., *Halictus* sp., *Pseudapis* sp., and *Sphecodes* sp. 

Bumble bees, which forage on *Lathyrus japonicus* [43] and *F. esculentum* [44], and megachilids that also forage on Apiaceae [45], are among the efficient pollinators for tomato. However, in our study neither the WM, which contained *L. sativus* and the Apiaceae *C. sativum* and *A. graveolense*, nor the SM which was dominated by *F. esculentum*, attracted any of these pollinators. The only presence of bumble bees was observed on *G. officinalis* that emerged late in the cropping season in the natural vegetation outside the designated plots. The capacity of *G. officinalis* to attract bumble bees, including *B. terrestris*, has also been reported by Montalva et al. [46]. 

The main pollinating insects attracted by the flowers of *L. sativus* in the WM were honeybees early in the season which are considered to contribute to the pollination of *L. sativus* [47], some syrphid flies and a few *Eucera* sp., which are known to prefer plants of the Fabaceae family. 

*Glebionis coronaria*, which provided a long-lasting flowering throughout the season, and *A. graveolens* attracted wild bees. Flowering of these species overlapped with that of the tomato crop and lasted until the fruit-set, providing an attractive habitat for potential pollinators of the crop. *Glebionis* spp. is known to attract wild bees and honeybees (M. Edwards pers. obs.). In our study, *G. coronaria* also attracted syrphids, *Odontomyia* sp. and other flies, as well as Lepidoptera. *Coriandrum sativum*, the second Apiaceae in the WM with intermediate flowering period and duration, also attracted wild bees and fewer honeybees. Studies in other countries have also reported that the main pollinating visitors of *C. sativum* were honeybees and wild bees of the Andrenidae, Halictidae, and Colletidae families (Algeria) [48], or honeybees and *Megachile* ssp. (Pakistan) [45].

*Fagopyrum esculentum*, which was the main flowering species in the SM, attracted fewer wild bee species (mainly *Andrena* spp. and *Halictus* spp.) but had significantly more visits than the WM, where the main flowering was provided from *C. sativum*, *A. graveolense* and *G. coronaria* in mid-June and *A. graveolense*, *G. coronaria* and *P. echioides* in mid-July. In addition, it attracted syrphids, other flies and Lepidoptera, as also reported by Thapa [49]. Previous studies have reported the capacity of *F. esculentum* to upgrade the floral resources of field margins for the benefit of pollinators and beneficial arthropods [29,50]. Although the species of pollinating insects visiting the flowers of *F. esculentum* differ in various parts of the world, in most cases, *A. mellifera* is reported as the main pollinator species [41]. Other wild bees foraging on the *F. esculentum* flowers, include *Bombus* spp. [41,44], *Xylocopa* spp., and Halictidae species, including *Halictus* spp. in Florida [41]. In our study, *F. esculentum* attracted both wild bees and honeybees (mid-July). At that time *P.* *tanacetifolia* was visited by wild bees. In other studies, *P.* *tanacetifolia* was visited by both honeybees and wild bees [23], or only honey bees [51]. This discrepancy might be explained by the generally low numbers of honeybees in the area at the time of *P. tanacetifollia* flowering (mid-July). The suitability of *P. tanacetifollia* species as a flower resource to honeybees and wild bees should be re-considered, according to some studies [52]. 

The NV plots had a plant diversity comparable to the sown mixtures, with species flowering early (*R. rugosum*, *A. altissima*) or later in the season (*S. atropurpurea*, *P. echioides*, *D. carota* and *C. arvensis*). However, the overall flowering provision was low and was reflected on the small number of the attracted species and visits of wild bees (*Andrena* spp., *Lasioglossum* spp.). An exception was for *R. rugosum*, which was foraged mainly by honeybees early in the season and a smaller number of wild bees later. *Scabiosa* spp. has been reported to attract wild bees and Lepidoptera, while *C. arvensis* can attract *Lasioglossum* sp. [23]. Nevertheless, these plant species should be managed with caution in a conservation plan that relies on natural vegetation in the field margins, since *C. arvensis* is a noxious weed [53] and *R. rugosum* has an invasive habit [54]. *Galega officinalis* that emerged in the surrounding area attracted *Bombus* sp., albeit later in the flowering of tomato. In a previous study, *G. officinalis* was among the main plant species that provided floral resources to *Bombus* sp. [55]. Since *Bombus* sp. are among the main pollinating insects for field-grown tomato, the presence of *Galega* sp. in the field margins of this crop is favorable. 

In our study, the presence of pollinators on tomato flowers was scarce during full flowering of the crop, regardless of the distance between the tomato rows and the sown mixture in mid-June, coinciding with the end of the sown field margins and the increasing flowering and the peak of *F. esculentum* in the summer mixture. Hence, flowers attractive to Hymenoptera pollinators at the sown margins did not affect visits of pollinators on tomato flowers and subsequently they neither distracted (no effect at the neighboring rows) nor increased pollinators in the tomato field.

The WM attracted an arthropod community (phytophagous insects, natural enemies) of greater diversity in comparison to the SM and the NV sites. It attracted more parasitoid families, with a larger number of individuals throughout the season, although the family sizes fluctuated with time. Overall, the flower cover diversity had only a weak correlation with the beneficial arthropod abundance but not their diversity. This was probably due to the large variability between the various field margin types (sown or with natural vegetation) which may have hindered the evidence of the positive relationship between beneficial arthropods and flowering plant diversity, confirmed in other studies [56].

The WM plots maintained parasitic wasps from 16 families, with Scelionidae and Eulophidae being the most abundant families in both the sown and natural vegetation margins, followed by Eurytomidae, Braconidae, and Ichneumonidae, depending also on time. Tetracambidae was found only in NV1 at the beginning of the season in May. Aphelinidae, including aphid parasitoids (*Aphelinus* spp.) and whitefly parasitoids (e.g., *Encarsia formosa*), was recorded mostly in NV2 in June at the end of flowering of *R. rugosum* and during the flowering of *C. intybus*. Trichogrammatidae was recorded only in NV2 in late-June, when scarce flowering from *C. intybus* and *C. arvensis* was present. This important parasitoid taxa includes species which attack major tomato pests, such as *H. armigera* and the South America tomato leafminer *Tuta absoluta* (Meyrick) (Lepidoptera: Gelechiidae) [57]. Ceraphronidae were mostly found both in the WM and NV in late June and Encyrtidae even later in mid-July. The two natural vegetation sites had similar overall flower cover, but different plant species present at the end of May when main flowering in NV1 was from *A. altissima* and *C. arvensis* and in NV2 from *R. rugosum*. The flowering of the SM, although practically coming from one species, *F. esculentum*, attracted parasitoids of 5–8 families. These were the same with the predominant and most abundant ones of the WM, at that time of the season. These results agree with Campbell et al. [41] who reported that 62 insect species from 16 families were flower visitors in *F. esculentum* fields, including parasitoids, predators, and pollinators, with the most common ones being parasitoid wasps of various arthropod pests; based on this evidence, they suggested the possible use of *F. esculentum* as a cover crop to enhance biological control of various pest arthropods within cropping systems or to augment local pollinator populations. Moreover, [51] reported other insect groups (e.g., Syrphidae, Sphecidae, Crabronidae, Vespidae, and Scoliidae) as common visitors of *F. esculentum*.

The beneficial effect of enhancing crop fields with nectar-producing flowering plants on parasitism rates and biological control of insect pests has been reported by many researchers [58,59,60]. Under the scope of functional biodiversity, the potential of the sown mixtures to attract natural enemies for the suppression of the crop pests has been acknowledged in several studies, many of them summarized by Balzan et al. [28]. Our results showed that the floral nectar resources provided in the flower margins, especially early in the season, can serve as a reservoir of parasitoids which would contribute to biological control against several pests that can attack tomato. 

The most abundant parasitoid family in both mixtures and natural vegetation, Scelionidae, includes egg parasitoids which have an efficient role in the biological control of pentatomids agricultural pests (Hemiptera: Pentatomidae) [61]. The flowers with narrow and tubular corollas with nectaries at the base such as the heads of the Asteraceae, which were the longest lasting flowering (*G. coronaria* in the WM, from early-May to mid-July) can provide nectar to parasitoids of Braconidae [62]. Both families include parasitoid wasps against *H. armigera*, *T. absoluta*, and Diptera leafminers (*Liriomyza* spp.) [63]. Among the numerous reviewed parasitoid species and families of *T. absoluta*, *Trichogramma* spp. (Trichogrammatidae) are of economic importance and are commercialized used as biological control agents [64]. It is noteworthy that *Trichogramma* spp. were found only in the NV2 site at the late-June sampling, when its relatively low flowering was provided mainly by *C. intybus* and *C. arvensis*. This was surprising because at that time the SM site was dominated by flowering *F. esculentum*, a known *Trichogramma*-attracting species [65] and also reported to increase the longevity and fecundity of Trichogrammatidae [66]. Romeis et al. [65] suggested that the small size of *Trichogramma* spp. renders them easily affected by turbulence, which affects their active flight more than larger species. This could potentially limit the effectiveness of spatially restricted types of habitat manipulation, such as the establishment of plant mixtures in field margins [67] and could have affected the presence of *Trichogramma* spp. in the sites we studied.

The high presence of Aphelinidae parasitoids on Brassicaceae is probably associated with the presence of host aphids on these plants (*R. rugosum*) [68]. Nevertheless, Pteromalidae and Ichneumonidae, present in the WM early in the season and Pteromalidae in the SM later in the season include several parasitoid species of aphids (Universal Chalcidoidea Database). Balzan et al. [28] highlighted the functional value of Apiaceae (*A. gravelonens*, *C. sativum*, *Foeniculum vulgare* Mill.) for several parasitoid wasps (Chalicidoidea, Ichneumonidae, Braconidae, Platygastroidea), whose abundance did not increase by the inclusion of species from Fabaceae, Asteraceae, Polygonaceae and Brassicaceae as expected because the dish-bowl flowers of Apiaceae are a more accessible sugar resource than the bell-funnel flowers of Fabaceae and the head-brush flowers of Asteraceae [39,40,62]. In our study, the relatively high presence of parasitoids towards the end of the season (mid-July) in the WM, when *C. sativum* flowering was ending, was probably maintained by the late flush of flowering in *G. coronaria* and *A. graveolense*, but also by the flowering of the weed *P. echioides*. According to Jana and Shekhawat [69], *C. sativum* and *A. graveolens* have a remarkable pest control impact when sown together, while *A. graveolens* is a good nectar resource for the egg parasitoid *Edovum plutteri* Grissel (Chalcididae) of the major pest of potato *Leptinotarsa decemlineata* Crawford (Coleoptera: Chrysomelidae) that can also attack tomato.

Both sown mixtures were habitats for spiders and generalist insect predators of the families Anthocoridae, Chrysopidae, Coccinellidae, Miridae and Syrphidae that prey on soft body insects such as aphids, whiteflies and thrips. Therefore, the plant species selected for the WM mixture could serve as banker plants for the control of these pests. Balzan et al. [28] argued that *A. gravelonens* and *C. sativum* can support the predator groups of Coccinelidae and Thomissidae (Araneae). Moreover, despite the presence of herbivores on the plants in the sown mixtures, there was no evidence of an increased infestation on tomato plants by aphids, Lepidoptera pests, or Heteroptera bugs, pointing to any disservices of the sown margins. Foraging of Lepidoptera tomato pests such as *T. absoluta* on nectar resources of the flower habitats is a concern [28], but Balzan and Wäckers [70], investigating the impact of flower strips with similar plant species synthesis, did not record an increase in Lepidoptera-caused crop damage. There was no evidence for the increase of the presence *Nezara viridula* (Linnaeus) (Hemiptera: Pentatomidae) or *Lygus* spp. in the sown mixtures, as found by Balzan et al. [28]. Similar studies have shown that wildflower strips can function as a trap crop for these pests [29] and less fruit damage was recorded in the crop adjacent to these strips [70].

## 4. Materials and Methods

### 4.1. Selection of Plant Species

Two plant mixtures, one with winter (WM) and one with summer species (SM) were composed to provide a diverse and long-lasting flower cover in the tomato field margin (Table 2). There is currently no seed house in Greece that would provide seeds of indigenous plant species. For this reason, most of the plant species were selected among those available in the Greek Gene Bank (GGB) of the Institute of Plant Breeding and Genetic Resources, Hellenic Agricultural Organization—ELGO DEMETER, Greece, that propagated them for the purposes of the project. Using indigenous genetic resources was imperative, to avoid genetic erosion of local populations from foreign seed material and to increase the likelihood of the mixture’s successful establishment. The final selection among the available plant species was based on criteria described before [23]. Briefly, the selected plants were dicotyledonous species with annual life cycle, recorded in the area, not listed as noxious weeds, and belong to a diverse range of families. In addition, we consulted literature data and results available from previous experiments on the success of species establishment and their field performance (flowering period and duration, attractiveness to pollinators or beneficial insects, relative vigor) for the final composition of the mixture and the relative analogy of each species in it. Based on the above, the WM included a total of seven plant species belonging to three families, as follows: Apiaceae (*Anethum graveolens* L., *Coriandrum sativum* L.), Asteraceae (*Glebionis coronaria* (L.) Cass. ex Spach, *Matricaria chamomilla* L., *Tagetes patula* L.) and Fabaceae *(Lathyrus sativus* L., *Vicia villosa* Roth.). Seeds of these species were provided by the GGB, except for *G. coronaria* which was collected from a spontaneous population in Attica, Greece. The SM included the indigenous Apiaceae *Petroselinum crispum* (Mill.) Johann Mihály Fuss (Fuss). However, since no other summer species was available at the time in the GGB, the SM included also two non-indigenous species widely regarded as highly attractive to honeybees, *Phacelia tanacetifolia* Benth. (Boraginaceae) and *Fagopyrum esculentum* Moench (Polygonaceae) whose seeds are available in the Greek market. 

### 4.2. Seed-Rate Calculation

The seed-rate per species in the mixtures was calculated taking into account the target plant density, the required percentage of each species in the mixtures and the estimated plant survival rate in the field, and seed parameters like the thousand grain weight and the germination percentage, following seed germination assays. The seed weight in grams for each species in a mixture (Ws) was the output of the following equation, modified from [23]:

$$\mathrm{Ws}\;=\;\mathrm{Ps}\;\times \left(\frac{1}{\mathrm{ESR}}\right)\times \left(\frac{1}{\mathrm{Pg}}\right)\times \;\mathrm{Tp}\;\times \;A\;\times \frac{\mathrm{TGW}}{1000}$$

where: Ps = Target percentage of plants per species, as presented in Table 1, ESR = estimated survival rate of plants, Pg = germination percentage from petri-dish assays, Tp = Total target number of plants/m^2^ (set at 100 plants/m^2^), A = sown area, and TGW = thousand grain weight (g). The relative proportion of the species in both mixtures is presented in Table 2.

### 4.3. Experimentation Site

**Figure 5:**
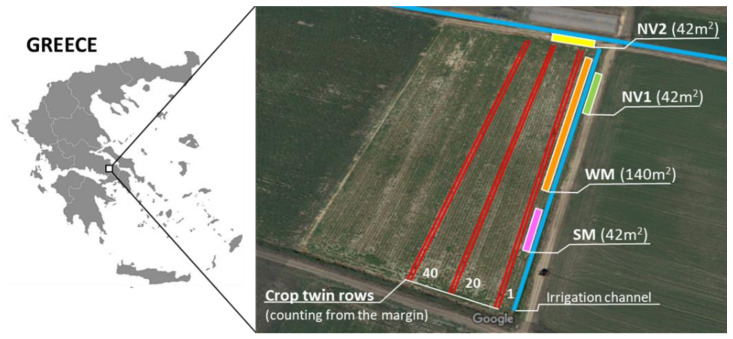
Experimentation site (38.501311, 22.918594) in Orhomenos, Viotia, central Greece and layout of the sown mixtures (winter WM and summer SM) and the two natural vegetation sites (NV1 and NV2) in the field margin of a processing tomato field. The red lines indicate the crop rows (1–2, 20–21 and 40–41 twin rows from the sown margin) where the presence of pollinators was recorded to assess for possible distraction effect from the sown flowering plants.

The experimentation site was a 2-ha field in the agricultural area of Orhomenos, Viotia (38.501311, 22.918594), one of the main areas in Greece where processing tomato is cultivated (Figure 5). The mixtures were sown in one of the field margins running parallel to a drainage/irrigation channel. Plot size was 14 m^2^ (7 m long × 2 m wide), with ten plots for the WM (total sown area 140 m^2^) and three plots for the SM (total sown area 42 m^2^). The control plots were selected in two separate sites with natural vegetation (NV1 and NV2) along the uncropped area next to the irrigation channel, to cater for the different arable weed communities present around the field. Each control site had three plots (total area per control site 42 m^2^).

Sowing of the mixtures was performed by hand, on 2 December 2014 (WM) and 7 May 2015 (SM), in separate parts of the field margin (Figure 5). The water collected during the winter and spring rains in the irrigation/drainage channel next to the sown field margin, maintained the soil moisture at adequate levels for seed germination and subsequent plant development, eliminating the need for additional irrigation. 

The crop was transplanted on 10 May 2015, in twin row sets (0.45 m between each row in the set, and 1.2 m between twin rows). The processing tomato was the joint-less variety H3402 (Heinz) with mid-maturity and medium plant size, commonly planted in the area. Irrigation, fertilizer and crop protection inputs were applied by the farmer, as required. 

### 4.4. Measurements

Measurements of flowering and attracted insects were performed during the main flowering period of the sown plants (WM from early-May to mid-July and SM at mid-June and mid-July), the control sites (NV1 and NV2 from early-May to mid-July), and the tomato crop (T, at mid and late-June), at the dates listed in Table 3.

The methodology was as described before [23] and adapted for the current study. More specifically, the total plant cover and flower cover (total and per species), were visually estimated and expressed as percentage cover/plot in all plots of the sown mixtures and the two control sites. Plant species were identified in situ or when necessary, in the lab using the botanical identification key Flora Europaea [71].

Hymenoptera pollinator visits on the flowers of the sown margins and the control plots, were recorded with visual observation of landings for 4′/plot between 10:30 and 14:30 h. The observations and corresponding counts refer to the foraging visits of pollinators and not their absolute numbers which could not be accurate due to their high abundance and their mobility from flower to flower during the observation time. The same observer recorded the pollinator visits on the flowers of all treatments throughout the sampling period to eliminate potential bias between different observers. Weather conditions which could affect insect populations and their flying (wind, temperature, cloud cover) were also recorded to ensure that measurements were performed near the ideal conditions of, wind up to 2 Beaufort, temperature in the range of 17–30 °C, and cloud cover of up to 50%. The actual weather conditions for the area before and during the experiment as recorded by a nearby weather station, are presented in Supplementary Figure S3. Measurements were conducted in five of the ten plots of the WM, in three plots of the SM and in three plots for each of the NV1 and NV2 sites. 

Hymenoptera pollinator visits were also recorded on the crop flowers with visual observation as before, at three sites based on the distance from the sown field margin, starting from the first two twin crop rows next to the margin and moving infield to the 20th-21st and 40th-41st twin rows (Figure 5). Each sampling site had three replications of approximately 14 m^2^ (2 twin rows, 7 m long). The number of crop plants and flowers were also recorded for each replication.

Wild bee specimens that required identification after the visual observation measurements, were captured with a sweeping net and were identified later in the lab. Identification of pollinators to genera was performed at BPI, Greece by Myrto Barda, based on the identification keys by Michener [72,73] and verified by M. Edwards, U.K.

Beneficial arthropods were recorded with suction sampling (1′/plot) using a modified leaf-blower (Echo ES-2400, 24 cm^3^, Kioritz Corporation, Tokyo, Japan) adapted as described in Stewart and Wright (1995) (Supplementary Figure S4). Measurements were conducted in the other five plots of the WM, three plots of the SM and three plots for each, NV1 and NV2 sites. The collected arthropod samples were kept in the freezer (−18 °C) and sorted according to family, genus, and species (where possible) under a stereo-microscope. Identification of the parasitoid taxa was performed at Alexandru Ioan Cuza University of Iaşi, Romania. Identification of Pteromalidae species was based on the identification keys by Graham [74]. Pollinators captured in the suction samples were also identified as mentioned above. 

### 4.5. Statistical Analysis

Flower synthesis of both mixtures and natural vegetation sites differed over time. Therefore, the data on the effect of mixture and sampling date on vegetation and flowering cover, as well as on pollinators, natural enemies, arthropod and parasitoid diversity (Shannon H index) were analyzed using two-way ANOVA (α = 0.05). Data on the number of beneficial insects (predators, parasitoids, total) and pollinators were transformed to their natural logarithm, to achieve better fitting to the assumptions of the analyses (homoscedacidity). Data on the flower cover percentage were arcsin transformed. When significant, means were separated using Tukey’s HSD test. When ANOVA showed no significant interaction, then comparisons among main effects were made. If ANOVA showed significant interaction, comparisons of the simple effects of each factor at the levels of the other factor were made. The statistical analyses were performed using the statistical package JMP (v7, SAS Institute Inc., Cary, NC, USA).

## 5. Conclusions

Selected flowering plants in the field margin of a processing tomato crop can attract wild bees and other pollinators, as well as beneficial arthropods and have a positive effect on their population abundance and diversity. Our results provide evidence that field margin management with selected plants from a range of families, including Asteraceae, Fabaceae, and Apiaceae, can benefit field-grown tomato, because it attracted wild bee species (e.g., *Lasioglossum* spp. and *Hylaeus* spp.) that can support the cross-pollination of tomato flowers, as well as the creation of a reservoir of parasitoids and predators that could contribute to the biological control tomato pests. The lack of any evidence for the potential distraction of pollinating insects from the processing tomato crop suggests that field margin management with selected flowering plants can be a sustainable approach in this crop. Future research should examine the possible effect of insect pollination on tomato fruit quality attributes and quantify the potential of the sown margins to support biological control of tomato pests.

## Figures and Tables

**Table 1:** Shannon diversity Index (*H*) of total arthropods and Hymenoptera parasitoids (mean ± s.e.m.) in 1′ suction samples/plot with sown selected flower plants or with natural vegetation at the margins of a processing tomato crop, from late-May to mid-July.

Field Margin	Shannon’s Diversity Index (*H*)											
	Total Arthropods						Hymenoptera Parasitoids					
	WT				Mean 21/5–15/7	Mean 17/6 &15/7	WT				Mean WT/Margin (21/5–15/7)	Mean WST/Margin (17/6 & 15/7)
			WST						WST			
	21/5	3/6	23/6	15/7			21/5	3/6	23/6	15/7		
WM	1.96 ± 0.06	1.91 ± 0.04	2.57 ± 0.08	2.60 ± 0.07	2.26 ± 0.08 A	2.58 ±0.05 A	1.77 ± 0.18	0.59 ± 0.34	1.35 ± 0.21	1.67 ± 0.09	1.34 ± 0.15 A	1.51 ± 0.12
NV1	1.51 ± 0.25	1.66 ± 0.29	2.46 ± 0.03	1.96 ± 0.16	1.90 ± 0.14 B	2.21 ±0.13 B	0.94 ± 0.23	0.23 ± 0.23	1.70 ± 0.22	0.84 ±0.26	0.93 ± 0.19 B	1.28 ± 0.24
NV2	1.80 ± 0.03	1.97 ± 0.18	2.40 ± 0.11	1.98 ± 0.11	2.04 ± 0.08 B	2.19 ±0.12 B	1.04 ± 0.04	0.66 ± 0.38	1.62 ± 0.14	0.89 ± 0.21	1.05 ± 0.15 AB	1.26 ± 0.20
SM			2.21 ± 0.26	2.16 ± 0.20		2.19 ±0.15 B			16.7 ± 3.4	12.0 ± 2.1		0.89 ± 0.30
Mean WT/date	1.79 ± 0.08 c	1.86 ± 0.09 c	2.49 ± 0.05 a	2.25 ± 0.11 b			1.34 ± 0.15 a	0.51 ±0.19 b	1.52 ± 0.12 a	1.23 ± 0.15 a		
Mean WST/date			2.43 ±0.07 b	2.23 ±0.09 a					0.99 ± 0.53	0.80 ± 0.41		
WT 2-way ANOVA	Mixture: F_2,32_ = 9.94, *p* = 0.0004; Date: F_3,32_ = 21.33, *p* < 0.0001; Mixture × Date: F_6,32_ = 1.99, *p* = 0.0966						Mixture: F_2,32_ = 3.53, *p* = 0.0413; Date: F_3,32_ = 10.16, *p* < 0.0001; Mixture × Date: F_6,32_ = 2.12, *p* = 0.0788					
WST 2-way ANOVA	Mixture: F_3,20_ = 5.57, *p* = 0.0060; Date: F_1,20_ = 6.55, *p* = 0.0187; Mixture × Date: F_6,20_ = 2.13, *p* = 0.1288						Mixture: F_3,20_ = 1.99, *p* = 0.1475; Date: F_1,20_ = 3.71, *p* = 0.0686; Mixture × Date: F_6,20_ = 2.46, *p* = 0.0928					

**Table 2:** Species selected for the mixtures of winter (WM) or summer (SM) dicotyledonous plants, sown in the margins of a processing tomato crop, and corresponding percentage of seed weight and number in the mixture.

		Seed Weight (%)		Seeds/Species (%)	
Family	Species	WM	SM	WM	SM
Apiaceae	*Anethum graveolens*	1.97		33	
	*Coriandrum sativum*	4		7	
	Petroselinum crispum *		17		54
Asteraceae	*Glebionis coronaria*	7			
	*Matricaria chamomilla* *	0.03		7	
	*Tagetes patula* *	5		31	
Fabaceae	*Lathyrus sativus*	80		13	
	*Vicia villosa*	2		1	
Polygonaceae	*Fagopyrum esculentum*		68		11
Boraginaceae	*Phacelia tanacetifolia*		15		36

* Plant species that did not contribute to the flowering of the mixtures.

**Table 3:** Measurement dates for (a) flower cover (F), Hymenoptera pollinator visits (H) and attracted beneficial arthropods (B) in the sown margin (with winter mixture WM, or summer mixture SM) and the natural vegetation sites (NV1 and NV2), and (b) for number of flowers and pollinator visits in the processing tomato crop (T).

	6-May	21-May	3-June	17-June	23-June	15-July
WM	F, P	F, P, B	F, P, B	F, P	B	F, P, B
SM				F, P	B	F, P, B
NV1	F, P	F, P, B	F, P, B	F, P	B	F, P, B
NV2	F, P	F, P, B	F, P, B	F, P	B	F, P, B
T					F, P	F, P

## Data Availability

The data that support the findings of this study are available from the corresponding authors (V.K., F.K.), upon reasonable request.

## Supplementary Materials

The following are available online at https://www.mdpi.com/article/10.3390/plants10051003/s1: Table S1. Mean percentage of flower cover (±s.e.m.) in the winter mixture (WM), the summer mixture (SM) or natural vegetation NV1 and NV2 sites, at the field margins of a processing tomato crop in five sampling dates (May–July 2015). Table S2. Mean number (±s.e.m.) of Hymenoptera pollinator visits/plot/4′ in the winter mixture (WM), the summer mixture (SM) or natural vegetation NV1 and NV2 sites, at the field margins of a processing tomato crop in five sampling dates (May–July 2015). Table S3. Pollinator genera and associated flowering in the sown mixtures and natural vegetation at the field margins of processing tomato crop. Table S4. Arthropod taxa recorded in 1′ suction samples/plot from the sown winter mixture (WM), summer mixture (SM) or natural vegetation NV1 and NV2 sites, at the field margins of a processing tomato crop in four sampling dates (May–July 2015). Table S5. Mean number (±s.e.m.) of Hymenoptera parasitoids and predators in 1′ suction samples/plot from the winter mixture (WM), the summer mixture (SM) or natural vegetation NV1 and NV2 sites, at the field margins of a processing tomato crop in four sampling dates (May–July 2015). Table S6. Parasitoid taxa (mean number (M) and Relative Abundance %) recorded in 1′ suction samples/m2 from the sown winter mixture (WM), summer mixture (SM) or natural vegetation NV1 and NV2 sites, at the field margins of a processing tomato crop in four sampling dates (May–July 2015). Figure S1. Overview of the sown field margin with the winter mixture (WM), during plant growth and flowering. Figure S2. Overview of the natural vegetation at the two sites (NV1 and NV2), separately and in relation to the sown margin and the crop, at different dates. Figure S3. Climatic conditions (temperature, precipitation, wind) in the experimentation area for the year 2015, during the sampling period. The sampling dates are indicated in the x-axis. Figure S4. Suction sampling device: A modified leaf-blower (Echo ES-2400) operating in reverse mode (suction) and fitted with a mesh bag to collect the insects.
