# Supplementary material for: Sown Wildflowers Enhance Habitats of Pollinators and Beneficial Arthropods in a Tomato Field Margin
Source: Plants (Basel). 2021 May 17;10(5):1003. doi: 10.3390/plants10051003 (PMC8156626; doi:10.3390/plants10051003)
Supplement: Supplementary file 1 [file plants-10-01003-s001.zip › plants-1187351-supplementary.pdf]

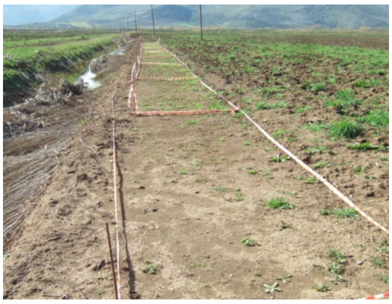
3/4/2015

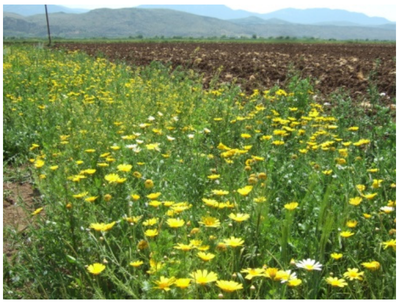
6/5/2015

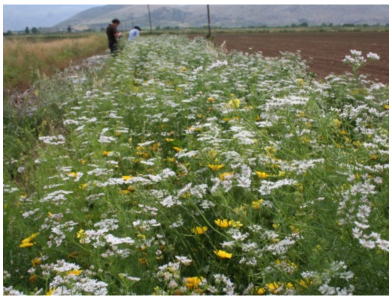
21/5/2015

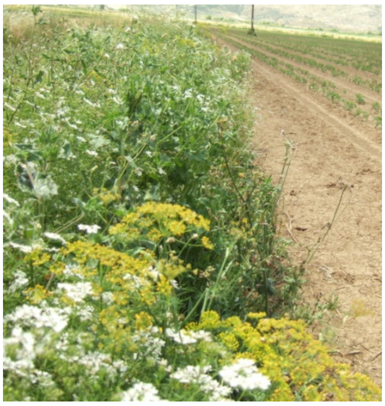
3/6/2015

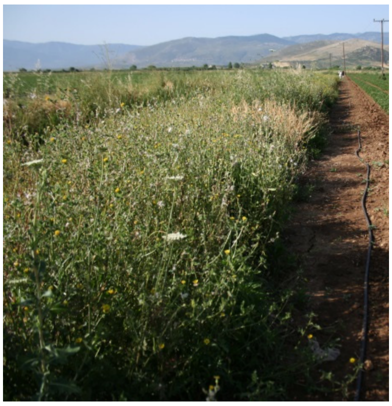
17/6/2015

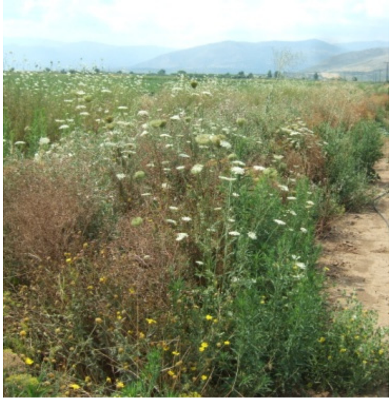
15/7/2015

Figure S1. Overview of the sown field margin with the winter mixture (WM), during plant growth and flowering.

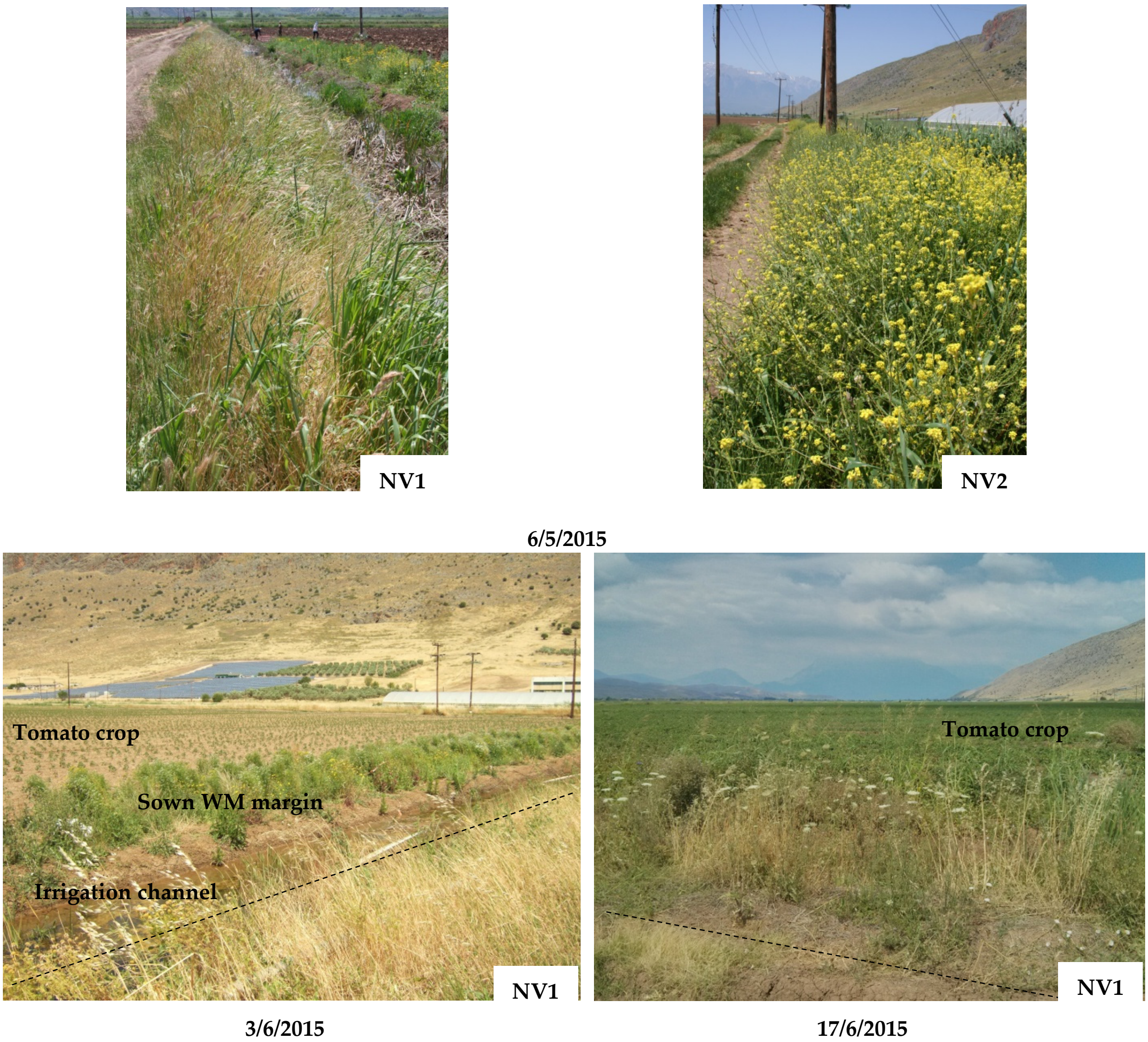

Figure S2. Overview of the natural vegetation at the two sites (NV1 and NV2), separately and in relation to the sown margin and the crop, at different dates.

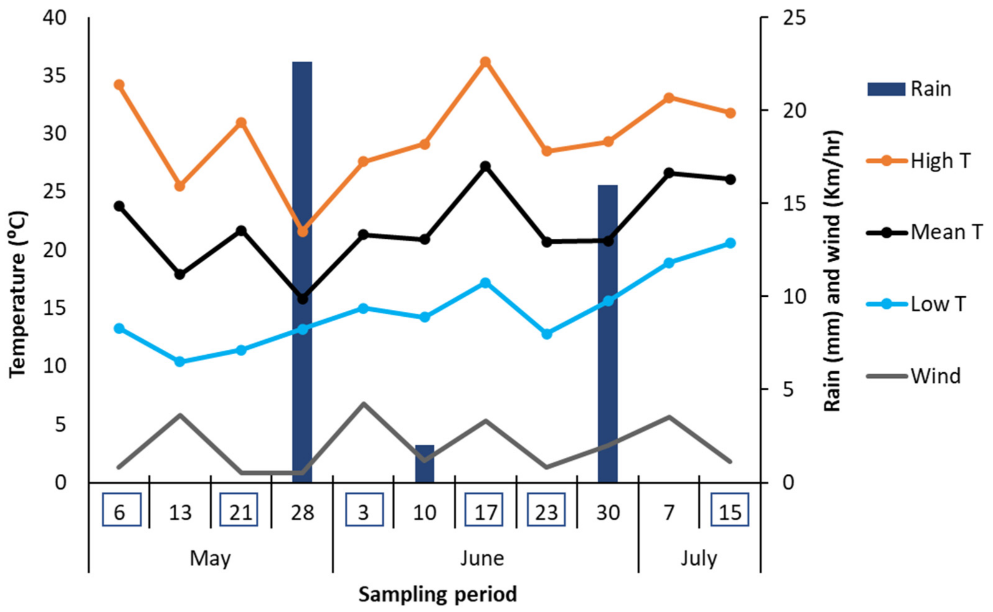

Figure S3. Climatic conditions (temperature, precipitation, wind) in the experimentation area for the year 2015, during the sampling period. The sampling dates are indicated in the x-axis.

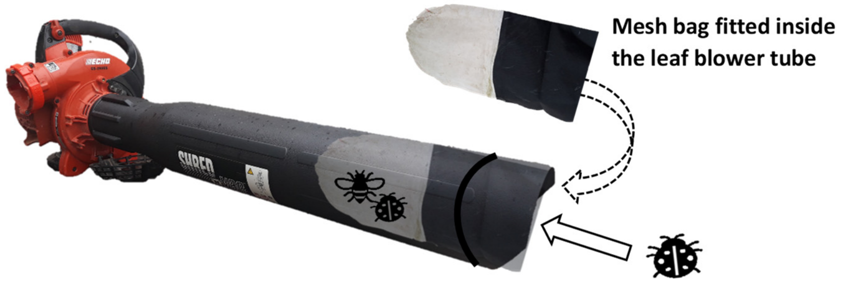

Figure S4. Suction sampling device: A modified leaf-blower (Echo ES-2400) operating in reverse mode (suction) and fitted with a mesh bag to collect the insects.

Table S1. Mean percentage of flower cover (±s.e.m.) in the winter mixture (WM), the summer mixture (SM) or natural vegetation NV1 and NV2 sites, at the field margins of a processing tomato crop in five sampling dates (May-July 2015).

	Mean flower cover (%)						
	WT					Mean WT/margin	Mean WST/margin
				WST			
**Field margin**	**6/5**	**21/5**	**3/6**	**17/6**	**15/7**	**(6/5-15/7)**	**(17/6 & 15/7)**
**WM**	81,5 ±4,2 *Aa*	86 ±3,9 *Aa*	77 ±4,4 *Aa*	41,7 ±1,2 *Ab*	39 ±2,6 *Ab*	65 ±3,3	40,4 ±1,4 B
**NV1**	5,0 ±0,1 *Bb*	20,0 ±2,9 *Ba*	3,7 ±1,8 *Bb*	5,7 ±0,3 *Bb*	8,3 ±1,7 *Bb*	8,3 ±1,7	6,5 ±1,2 C
**NV2**	63,3 ±4,4 *Aa*	20,0 ±5,0 *Bb*	2,0 ±0,1 *Bb*	4,7 ±0,9 *Bb*	16,7±9,3 *Bb*	21,5 ±6,2	11,2 ±4,8 C
**SM**				74 ±17,7	55 ±10,8		78,3 ±6,5 A
**Mean WT/date**	63,8 ±4,3	61,3 ±6,9	49,2 ±27,3	28 ±7,6	29,1 ±7,2		
**Mean WST/date**				38,1±3 a	34,7 ±2,3 b		
**WT 2-way ANOVA**	Mixture: $F_{2,52}$ = 151.12, p < 0.0001; Date: $F_{3,52}$ = 44.49, p < 0.0001; Mixture*Date: $F_{6,52}$ =10.60, p < 0.0001						
**WST 2-way ANOVA**	Mixture: $F_{3,20}$ = 21.10, p < 0.0001; Date: $F_{1,20}$ = 5.73, p = 0.0266; Mixture*Date: $F_{3,20}$ = 1.38, p = 0.2782						

Two-way ANOVA (treatment and sampling date) on transformed data (arcsin). Capital letters indicate significant differences between treatments while small letters indicate significant differences between assessment dates. Comparison between main effects is depicted with normal letters, while comparisons among simple effects with italics (Tukey HSD).

WM: winter mixture; NV1: natural vegetation at site 1; NV2: natural vegetation at site 2; SM: summer mixture; WT: winter treatments (WM, NV1 & NV2, from 6/5 to 15/7); WST: winter and summer treatments (WM, SM, NV1 & NV2 for 23/6 & 15/7).

Table S2. Mean number (±s.e.m.) of Hymenoptera pollinator visits/plot/4′ in the winter mixture (WM), the summer mixture (SM) or natural vegetation NV1 and NV2 sites, at the field margins of a processing tomato crop in five sampling dates (May-July 2015).

	Total pollinators							Wild bees						
	WT					Mean WT/margin	Mean WST/margin (17/6 & 15/7)	WT					Mean WT/margin	Mean WST/margin (17/6 & 15/7)
				WST							WST			
Field margin	6/5	21/5	3/6	17/6	15/7	(6/5-15/7)	15/7)	6/5	21/5	3/6	17/6	15/7	(6/5-15/7)	15/7)
**WM**	11,8 ±2,4 *Aab*	19,6 ±3,8 *Aa*	11,4 ±2,1 *Aab*	5,4 ±1,3 *Abc*	3,6 ±1,2 *Ac*	10,4 ±1.5	4.5 ±0.9 B	4.4 ±1.8	16.2 ±2.3	10.8 ±1.9	5.4 ±1.3	3.6 ±1.2 A	8.1 ±1.2 A	4.5 ±0.9 B
**NV1**	0.0 ±0.0 *Ba*	4.0 ±3,1 *Ba*	5.0 ±3,2 *ABa*	0,3 ±0,3 *Aa*	1.0 ±0,6 *ABa*	2,1 ±0,9	0.3 ±0.2 C	0.0 ±0.0	4.0 ±3.0	5.0 ±3.2	0.3 ±0.3	1.0 ±0.6 B	2.1 ±0.9 B	0.3 ±0.2 C
**NV2**	12,3 ±3,8 *Aa*	3,3 ±0,3 *Bab*	0.0 ±0.0 *Bb*	3,7 ±2,7 *Aab*	0,3 ±0,3 *Bb*	3.9 ±1.4	2.3 ±1.4 BC	0.0 ±0.0	3.0 ±0.0	0.0 ±0.0	3.7 ±2.7	0.3 ±0.3 B	1.4 ±0.6 B	2.3 ±1.4 BC
**SM**				34.0 ±6.3	8.0 ±1.1		21.0 ±6.5 A				33.7 ±6.6	8.0 ±1.1		20.8 ±6.4 A
**Mean WT/date**	8..7 ±2.2	10.9 ±3.1	6.5 ±1.9	3.5 ±1.1	2.0 ±0.7			2.0 ±0.7B	9.3 ±2.3 a	6. 3±1.8 ab	3.5 ±1.1 ab	2.2±0.7ab		
**Mean WST/date**				10.9 ±3.3 a	3.3 ±0.9 b						10.8 ±3.3 a	3.3 ±0.9 b		
**WT 2-way ANOVA**	Mixture: $F_{2,40}$= 32.42, p < 0.001; Date: $F_{4,40}$ = 5.52, p=0.0012; Mixture*Date: $F_{8,40}$ = 4.46, p=0.0006							Mixture: $F_{2,40}$= 31.62, p < 0.0001; Date: $F_{4,40}$ = 6.477, p =0.0004; Mixture*Date: $F_{8,40}$ = 1.69, p = 0.132						
**WST 2-way ANOVA**	Mixture: $F_{3,20}$ = 21.10, p < 0.0001; Date: $F_{1,20}$ = 5.73, p = 0.0266; Mixture*Date: $F_{3,20}$ = 1.38, p = 0.2782							Mixture: $F_{3,20}$ = 20.90 p < 0.0001; Date: $F_{1,20}$ = 5.64, p = 0.0277; Mixture*Date: $F_{3,20}$ = 1.38, p = 0.2893						

Two-way ANOVA (treatment and sampling date) on transformed data (ln(x+1)). Capital letters indicate significant differences between treatments while small letters indicate significant differences between assessment dates. Comparisons between main effects are depicted with normal letters, while comparisons among simple main effects with italics (Tukey HSD). WM: winter mixture; NV1: natural vegetation at site 1; NV2: natural vegetation at site 2; SM: summer mixture; WT: winter treatments (WM, NV1 & NV2, from 6/5 to 15/7); WST: winter and summer treatments (WM, SM, NV1 & NV2 for 23/6 & 15/7).

Table S3. Pollinator genera and associated flowering in the sown mixtures and natural vegetation at the field margins of processing tomato crop.

Family	Genus	Associated flowering plants*	Number of specimens**
Andrenidae	*Andrena* spp., at least 5 morphospecies	*Calendula* sp., *Capsella* sp., Asteraceae, *Fagopyrum esculentum*, WM, SM, NV	18 (6 ♂, 12 ♀); net (11); suction (7)
Apidae	*Apis mellifera*	WM, SM	1 (♀); suction
	Eucera sp.	*Lathyrus sativus*	4 (♀); net
Colletidae	*Colletes* sp.	WM, SM	4 (2 ♂, 2 ♀); net (1); suction (3)
	Hylaeus spp., 5 morphospecies	WM, SM	5 (♀); net (1); suction (4)
	Hylaeus cornutus	na	1 (♀); net
Halictidae	*Halictus* spp., at least 3 morphospecies	WM, *Coriandrum sativum*, *Fagopyrum esculentum*	4 (♀); net
	Lasioglossum spp., at least 5 morphospecies	Asteraceae, *Glebionis coronaria*, *Coriandrum sativum*, *Sinapis* sp., *Anethum graveolens*, *Picris echioides*, WM, SM	28 (3 ♂, 25 ♀); net (11); suction (17)
	Pseudapis sp.	WM	2 (♂); suction
	Sphecodes spp., at least 3 morphospecies	*Coriandrum sativum*, WM, SM	5 (2 ♂, 3 ♀); net (2); suction (3)

* WM= winter mixture; SM= summer mixture; NV= natural vegetation; na: not available; sp. in parenthesis indicates the pollinator morphospecies.

** net= net sampling, suction = suction sampling

Table S4. Arthropod taxa recorded in 1′ suction samples/plot from the sown winter mixture (WM), summer mixture (SM) or natural vegetation NV1 and NV2 sites, at the field margins of a processing tomato crop in four sampling dates (May-July 2015).

CLASS	Order	Family, Genus, Species	21/05/2015			03/06/2015			23/06/2015				15/07/2015			
			WM	NV1	NV2	WM	NV1	NV2	WM	SM	NV1	NV2	WM	SM	NV1	NV2
INSECTA	Coleoptera	Cantharidae	4	0	0	0	0	0	0	0	0	0	0	0	0	0
		Coccinellidae	2	1	3	0	4	4	8	3	1	1	1	5	2	1
		Other	39	3	37	16	3	4	15	18	20	4	14	21	3	5
	Dermaptera		0	0	0	0	0	0	0	0	22	0	0	0	0	0
	Diptera	Syrphidae	5	0	0	3	1	1	1	3	0	0	3	3	1	0
		Other beneficials	12	0	5	2	0	0	3	0	3	0	2	1	1	0
		Flies and other	577	154	121	160	57	28	52	53	83	44	48	9	10	9
	Hemiptera	Anthocoridae	9	0	0	16	0	1	12	9	3	0	8	6	2	0
		Aphididae	67	3	5	51	15	43	29	2	43	10	8	1	4	7
		Cicadellidae	14	13	12	1	6	5	34	10	13	15	14	49	6	10

		Lygaeidae	5	0	0	0	0	0	96	18	16	4	90	8	11	12
		Miridae	24	0	0	2	1	0	55	2	1	0	5	9	0	0
		Nabidae	1	0	3	0	0	0	0	1	0	3	0	1	0	0
		Reduviidae	0	0	0	0	0	0	0	0	1	0	0	0	0	0
		Pentatomidae	2	2	2	5	1	7	25	2	4	7	62	15	5	10
		Psyllidae	1	5	12	0	3	0	0	1	0	0	0	0	0	0
		Tingidae	0	0	0	0	1	0	0	0	0	0	0	0	0	0
		Other	2	1	4	8	3	8	18	2	11	8	63	5	5	6
	Hymenoptera	Total parasitoids	150	31	29	26	3	12	225	20	66	38	278	14	36	59
		Total pollinators	13	0	1	19	1	1	2	7	0	0	1	0	0	0
		Formicidae	12	20	152	1	23	27	21	1	23	51	20	0	72	88
		Vespididae	0	0	0	3	0	0	1	0	0	1	1	0	0	0
	Lepidoptera		2	0	1	0	0	1	3	0	9	8	4	2	0	2
	Neuroptera	Chrysopidae	28	1	2	2	0	0	3	2	1	2	1	0	0	1
	Odonata		2	0	1	0	0	0	1	0	0	0	0	1	0	0
	Orthoptera		0	0	0	0	0	1	0	0	0	0	0	0	0	3
	Psocoptera		2	0	0	0	0	0	0	0	0	0	0	0	0	0
	Thysanoptera	*Aelothrips* sp.	126	0	2	49	1	1	15	37	8	3	5	5	0	0
		Other	29	2	7	14	1	28	18	10	40	2	43	2	6	1
ARACHNIDA	Araneae		15	8	5	23	6	7	70	11	28	31	83	8	21	27

Table S5. Mean number (±s.e.m.) of Hymenoptera parasitoids and predators in 1′ suction samples/plot from the winter mixture (WM), the summer mixture (SM) or natural vegetation NV1 and NV2 sites, at the field margins of a processing tomato crop in four sampling dates (May-July 2015).

	**Hymenoptera parasitoids**						**Predators**					
	**WT**				**Mean WT/margin**	**Mean WST/margin**	**WT**				**Mean WT/margin**	**Mean WST/margin**
			**WST**						**WST**			
**Field margin**	**21/5**	**3/6**	**23/6**	**15/7**	**(21/5-15/7)**	**(17/6 & 15/7)**	**21/5**	**3/6**	**23/6**	**15/7**	**(21/5-15/7)**	**(17/6 & 15/7)**
**WM**	33.8 ±6.7	5.8 ±3.1	44.6 ±8.1	56.4 ±6.8	35.1 ±5.2 A	50.5 ±5.3 A	41.8 ±8.3	13.8 ±3.2	47.2 ±8.5	59.6 ±5.8	40.6 ±4.9 A	53.4 ±5.3 A
**NV1**	10.3 ±3.2	1.0 ±0.6	23.0 ±7.0	12.7 ±2.0	11.7 ±2.9 B	17.8 ±4.0 B	9.0 ±2.1	4.0 ±2.3	28.3 ±3.8	13.7 ±6.4	13.6 ±3.2 B	20.7 ±4.8 B
**NV2**	9.7 ±3.2	4.3 ±1.2	12.7 ±2.3	19.7 ±2.9	11.6 ±2.0 B	16.1 ±2.3 B	12.0 ±0.6	7.3 ±2.7	23.3 ±5.5	16.0 ±1.1	14.7 ±2.2 B	19.7 ±3.0 B
**SM**			7.3 ±4.3	4.7 ±2.2		6.0 ±2.2 C			16.7 ±3.4	12.0 ±2.1		14.3 ±2.1 B
**Mean WT/date**	20.8 ±4.8 a	4.1 ±1.5 b	30.0 ±5.8 a	34.4 ±7.0a			24.7 ±6.1 a	9.4 ±2.1 b	35.5 ±5.2 a	35.0 ±7.7a		
**Mean WST/date**			25.0 ±5.3	28.1 ±6.5					31.5 ±4.6	30.1 ±6.5		
**WT 2-way ANOVA**	Mixture: $F_{2,32}$ = 10.48, p = 0.0003; Date: $F_{3,32}$ = 22.28, p < 0.0001; Mixture*Date: $F_{6,32}$ = 1.37, p = 0.2566						Mixture: $F_{2,32}$ = 20.44, p = 0.0001; Date: $F_{3,32}$ = 13.61, p < 0.0001; Mixture*Date: $F_{6,32}$ = 1.05, p = 0.4113					
**WST 2-way ANOVA**	Mixture: $F_{3,20}$ = 17.72, p < 0.0001; Date: $F_{1,20}$ = 0.05, p = 0.8187; Mixture*Date: $F_{6,20}$ = 0.66, p = 0.5887						Mixture: $F_{3,20}$ = 14.00, p < 0.0001; Date: $F_{1,20}$ = 3.66, p = 0.0699; Mixture*Date: $F_{6,20}$ = 2.64, p = 0.0778					

Two-way ANOVA (treatment and sampling date) on transformed data (ln(x+1)). Capital letters indicate significant differences between treatments while small letters indicate significant differences between assessment dates. WM: winter mixture; NV1: natural vegetation at site 1; NV2: natural vegetation at site 2; SM: summer mixture WT: winter treatments (WM, NV1 & NV2, from 21/5 to 15/7); WST: winter and summer treatments (WM, SM, NV1 & NV2 for 23/6 & 15/7).

Table S6. Parasitoid taxa (mean number (M) and Relative Abundance %) recorded in 1' suction samples/m^2 from the sown winter mixture (WM), summer mixture (SM) or natural vegetation NV1 and NV2 sites, at the field margins of a processing tomato crop in four sampling dates (May-July 2015).

		**21/5**						**3/6**						**23/6**								**15/7**							
		**WM**		**NV1**		**NV2**		**WM**		**NV1**		**NV2**		**WM**		**NV1**		**NV2**		**SM**		**WM**		**NV1**		**NV2**		**SM**	
**Superfamily**	**Family**	M	%	M	%	M	%	M	%	M	%	M	%	M	%	M	%	M	%	M	%	M	%	M	%	M	%	M	%
Ceraphronoidea	Ceraphronida	1	2	0	0	0	0	0	0	0	0	0	0	1	3	2	11	1	11	0	0	0	0	0	3	0	0	0	0
	Megaspilidae	0	0	1	7	0	3	0	0	0	0	0	0	0	0	0	2	0	0	0	0	0	0	0	0	0	0	0	0
Chalcidoidea	Aphelinidae	0	0	0	0	0	0	0	0	0	0	2	42	1	2	1	3	1	11	0	5	0	0	0	0	0	0	0	0
	Encyrtidae	0	0	0	0	0	0	0	0	0	0	0	0	3	6	0	0	1	8	0	5	12	22	1	8	2	12	1	14
	Eulophidae	13	44	4	36	3	35	1	15	0	0	1	17	11	24	5	24	4	29	1	10	13	23	1	11	3	14	1	28
	Eupelmidae	0	0	0	0	0	0	0	4	0	0	0	0	1	2	0	0	0	0	0	0	3	5	0	0	0	0	0	0
	Eurytomidae	0	1	0	0	0	0	2	39	0	0	0	0	12	27	0	0	0	0	1	20	5	9	0	3	1	3	0	0
	Mymaridae	0	1	0	0	0	3	0	0	0	0	0	0	0	0	2	11	0	3	1	10	2	3	1	8	0	2	1	14
	Pteromalidae	4	12	0	0	0	0	1	12	0	0	0	8	1	2	1	5	0	0	1	10	1	1	0	0	0	0	0	0
	Tetracampida	0	0	0	3	0	0	0	0	0	0	0	0	0	0	0	0	0	0	0	0	0	0	0	0	0	0	0	0
	Torymidae	1	2	0	0	0	0	0	0	0	0	0	0	0	0	0	0	0	0	0	0	0	1	0	0	0	2	0	0
	Trichogrammaidae	0	0	0	0	0	0	0	0	0	0	0	0	0	0	0	0	1	5	0	0	0	0	0	0	0	0	0	0
Cynipoidea		1	3	0	3	0	3	0	0	0	0	0	0	0	0	0	0	0	0	0	5	0	0	0	0	0	0	0	0
Diaprodoidea	Diapriidae	0	1	0	0	0	0	0	0	0	0	0	0	0	0	0	0	0	0	0	0	0	0	0	0	0	0	0	0
Ichneumonoidea	Braconidae	2	5	1	7	1	7	0	8	0	33	0	0	1	2	2	9	1	5	0	0	3	5	0	0	0	0	1	21
	Ichneumonida	2	7	0	0	1	7	0	0	0	0	0	0	0	0	0	0	0	0	0	0	0	0	0	0	0	0	0	0
Platygastroidea	Platygastridae	0	1	0	0	0	0	0	0	0	0	0	0	0	0	1	3	0	3	0	0	0	0	0	0	0	0	0	0
	Scelionidae	6	21	5	45	4	41	1	23	1	67	1	33	14	31	7	33	3	26	2	35	16	29	8	67	13	68	1	21
**Total mean**		**30**		**10**		**10**		**5**		**1**		**4**		**45**		**22**		**13**		**7**		**56**		**12**		**20**		**5**	
